# In Vitro Toxicity of Bone Graft Materials to Human Mineralizing Cells

**DOI:** 10.3390/ma15051955

**Published:** 2022-03-06

**Authors:** Fan Yang, Kao Li, Shi Fu, Michael Cuiffo, Marcia Simon, Miriam Rafailovich, Georgios E. Romanos

**Affiliations:** 1 Department of Materials Science and Chemical Engineering, Stony Brook University, Stony Brook, New York, NY 11794-2275, USA; fan.yang.2@stonybrook.edu (F.Y.); kao.li@stonybrook.edu (K.L.); shi.fu@stonybrook.edu (S.F.); michael.cuiffo@stonybrook.edu (M.C.); miriam.rafailovich@stonybrook.edu (M.R.); 2 Department of Oral Biology and Pathology, School of Dental Medicine, Stony Book University, Stony Brook, New York, NY 11794-8702, USA; marcia.simon@stonybrook.edu; 3 Department of Periodontology, School of Dental Medicine, Stony Brook University, Stony Brook, New York, NY 11794-8700, USA

**Keywords:** collagen, dental pulp stem cells, hydroxyapatite, osteosarcoma, Raman spectrum analysis, tungsten

## Abstract

Bone graft materials from synthetic, bovine, and human sources were analyzed and tested for in vitro cytotoxicity on dental pulp stem cells (DPSCs) and osteosarcoma cells (Saos-2). Raman spectroscopy indicated significant amounts of collagen only in human bone-derived materials, where the mineral to protein ratio was 3.55 ± 0.45, consistent with bone. X-ray fluorescence revealed tungsten (W) concentrations of 463 ± 73, 400 ± 77, and 92 ± 42 ppm in synthetic, bovine, and human bone chips, respectively. When these chips were added to DPSCs on tissue culture plastic, the doubling times after two days were the same as the controls, 16.5 ± 0.5 h. Those cultured with synthetic or bovine chips were 96.5 ± 8.1 and 25.2 ± 1.4 h, respectively. Saos-2 was more sensitive. During the first two days with allogeneic or bovine graft materials, cell numbers declined. When DPSC were cultured on collagen, allogeneic and bovine bone chips did not increase doubling times. We propose cytotoxicity was associated with tungsten, where only the concentration in human bone chips was below 184 ppm, the value reported as cytotoxic in vitro. Cells on collagen were resistant to bone chips, possibly due to tungsten adsorption by collagen.

## 1. Introduction

Bone grafting techniques are very common in surgical dentistry for rehabilitation of the missing dentition using dental implants. A variety of bone grafting materials and techniques have been introduced and utilized and show long-term success [1]. However, there is a high number of clinical conditions when the bone particles during the drilling for implant placement are not consolidated with the surrounding bone, and, therefore, additional (supplemental) grafting is required. The xenografts have been utilized in clinical practice demonstrating a high survival rate of dental implants compared to the autogenous bone grafts, which are associated with a high resorption rate [2]. There is no consensus when grafting materials are healed in the grafted site, and a variety of loading protocols has been recommended to stimulate bone growth [3,4].

Recent studies are evaluating the impact of compressive forces of calvaria defects in rabbits [5] and the extraction sites after grafting in dogs [6]. There is a positive impact on bone formation if particles are compressed, and no consistent evidence about the role of the bone substitutes used to fill critical size defects (CSDs) in rabbit calvariae to enhance defect closure [7,8]. However, there is a need for evaluation of the osteogenic potential of bone grafting materials and potential toxicity in vitro to improve this kind of grafting clinical outcomes. Specifically, bone chips are commonly used as fillers for strengthening dental implants and facilitating bone integration. Bone chips obtained from animal sources, though they are known to contain metal impurities, mostly from their feed [9,10,11,12]. A common impurity associated with bone is tungsten, which recent in vivo studies have demonstrated toxicity in mice that were exposed to concentrations as low as 15 ppm in their drinking water [13] and have long been suspected of toxicity in humans [14]. Here we analyzed the concentration of tungsten in commercially available bone chips and investigated the short-term effects on human dental pulp stem cells, as well as osteosarcoma bone cells where the cells were cultured on tissue culture plastic as well as on collagen, which is a major component of the natural extracellular matrix of bone [15].

## 2. Materials and Methods

### 2.1. Bone Graft Materials

Three different groups of commercially available bone grafting materials were used: Bio-Oss^®^ Cancellous Small Granules was from Geistlich Biomaterials (Wolhusen, Switzerland) and labeled as bovine mineral graft material (BMGM), Puros^®^ Cortico-Cancellous Particulate Allograft was from Zimmer Biomet (Palm Beach Gardens, FL, USA) and labeled as allograft graft material (AGM), and Symbios OsteoGraf^®^ LD-300 was from Dentsply Sirona (Charlotte, NC, USA) and labeled as synthetic graft material (SGM). In vivo, the bone chips are packed tightly.

### 2.2. Cells and Cell Culture

Dental pulp cells (DPSCs), strain 13, were isolated at the Stony Brook School of Dental Medicine from discarded third molar teeth under IRB exemption for de-identified surgical waste (CORIHS Project ID: 20076778, approval #00000125). These cells have previously been shown to promote mineralization on banded collagen and to express markers of osteogenesis (collagen 1, BSP, OPN) when grown on titania coated surfaces and induced [15]. Osteosarcoma cells, Saos-2, were purchased from ATCC (Manassas, VA, USA).

For expansion, DPSCs were plated at 7000 cells/cm^2^ and cultured in a growth medium comprising alpha minimal essential medium (α-MEM) (Gibco, Thermo Fisher Scientific, Waltham, MA, USA) supplemented with 10% fetal bovine serum (FBS) (Gibco, Thermo Fisher Scientific, Waltham, MA, USA), and 200 μM L-ascorbic acid 2-phosphate (Sigma-Aldrich, St. Louis, MO, USA). In order to harvest cells, cultures were washed 2–3 times with Ca, Mg-free phosphate-buffered saline and then incubated with Trypsin/EDTA (Gibco, Thermo Fisher Scientific, Waltham, MA, USA) for 5–10 min at 37 °C. Following cell detachment, trypsin was neutralized with serum-containing growth medium, and cells were collected by centrifugation and resuspended in growth medium.

For experiments, DPSC growth medium was supplemented with 10 mM β-glycerol phosphate (Sigma-Aldrich, St. Louis, MO, USA) (M-DPSC) and, where indicated, was further supplemented with 10^−8^ M dexamethasone (DEX) (Sigma-Aldrich, St. Louis, MO, USA); DEX is a common steroid that has been used to enhance DPSC differentiation [16]. For expansion and experiments with Saos-2 cells, low glucose Dulbecco’s Modified Eagle Medium (DMEM) (Gibco, Thermo Fisher Scientific, Waltham, MA, USA) supplemented with 10% FBS (M-SAOS) was used.

### 2.3. X-ray Fluorescence (XRF)

The bone chip samples were placed directly on 4 cm^2^ diameter, 5 μ thick, polypropylene film discs obtained from thin XRF film rolls (Spex, SamplePrep, Metuchen, NJ, USA) and analyzed directly on the films. XRF spectroscopy was performed on a Niton XL3t GOLDD + handheld XRF (Thermo Fisher Scientific, Waltham, MA, USA) using Niton Data Transfer Software (version 8.4.1.1) in mining mode. The data plotted represent the average of three trials.

### 2.4. Raman Spectroscopy

Raman spectra of bone graft materials were obtained using a Confocal Raman Microspectrometer (InVia, Renishaw, West Dundee, IL, USA) with a laser source of 514 nm at 1800 line/mm grating. Peak area was analyzed by WiRE 4.1 software.

### 2.5. Cell Plating and Bone Graft Materials Exposure

For experiments, DPSCs and Saos-2 were plated separately onto 24-well plates at a density of 5000 cells/cm^2^. Each well received a total of 1 mL of medium (M-DPSC for DPSCs and M-SAOS for Saos-2 cells), after which cultures were transferred to a humidified incubator with 5% CO_2_ and a temperature of 37 °C. Twenty-four (24) hours after plating, bone graft materials were added to the cell cultures at 16.7 mg/well, and the cultures returned to the incubator. Since the bone chips scattered light, Anwar [17] showed that optical imaging was difficult at high concentrations. We chose a concentration where we could still obtain good quality images using the EVOS microscope (Thermo Fisher Scientific, Waltham, MA, USA). In vivo, the bone chips are packed tightly, and hence this value is lower than that to which the cells would be exposed in vivo.

### 2.6. Cell Imaging and Cell Counting

Cells were fixed in 3.7% of formaldehyde (Sigma-Aldrich) for 15 min, permeabilized in 0.2% Triton X-100 (Sigma-Aldrich) for 7.5 min, washed twice with phosphate-buffered saline, and then stained with Alexa Fluor (AF) 488 Phalloidin (Molecular Probes, Eugene, OR, USA) to visualize actin filaments and with 4′,6-diamidino-2-phenylindole dihydrochloride (DAPI) (Sigma-Aldrich) to visualize nuclei. Stains were added according to the manufacturer’s protocols, and cells were imaged and counted using an EVOS FL Auto Cell Imaging System (Invitrogen, Waltham, MA, USA). The images shown are a superposition of the two channels. The scale bars, as shown, correspond to actual magnifications obtained from different lenses. In the lower magnification images, a composite of multiple images produced by the EVOS software (Thermo Fisher Scientific, Waltham, MA, USA) is shown.

### 2.7. Conditioned Media

Conditioned media were prepared by incubating M-DPSC and M-SAOS at 37 °C in 5% CO_2_ with bone graft materials (16.7 mg/mL) for 48 h at 37 °C. Bone graft materials were removed by centrifugation at 850 rpm for 5 min, and each supernatant was filtered through 0.02 μm filters. The filtered media were labeled as conditioned media (CM-DPSC and CM-SAOS). In order to prepare control media, the same process was carried out using M-DPSC and M-SAOS but incubated without the addition of bone graft materials.

### 2.8. Collagen Substrate

A 1 mg/mL collagen solution (EMD Millipore) containing 10% FBS (HyClone, Logan UT), 0.2% Na_2_CO_3_ (Sigma-Aldrich), DMEM and 1.7 mM L-glutamine (Gibco) was prepared at 4 °C with pH adjusted to 7.1 using 1N NaOH (Sigma-Aldrich). A total of 0.5 mL was added to each well of a 24-well plate used for the experiment. The plate was kept at room temperature for 45 min prior to the addition of 1 mL of medium and transferred to a humidified incubator with 5% CO_2_ at 37 °C for 24 h before cell plating.

### 2.9. Statistical Analysis

All experiments were performed in at least triplicates. The results are presented as mean ± SD. Statistical analysis was carried out using Version 8 of Graphpad Prism (GraphPad Software, San Diego, CA, USA). The errors of each data point were obtained from a minimum of three independent replicate tests, from where the mean and standard deviations were calculated. Comparative measurements between independent data sets were performed using unpaired t-test analysis with Welch’s correction or unequal variances t-test, where statistical significance was considered only for those having values of *p* < 0.05.

### 2.10. Image Data Collection

The cells were imaged using an Evos Auto-FL cell imaging system with two frequency cubes for green and blue fluorescence. The images shown are a superposition of the two channels. The scale bars, as shown, correspond to actual magnifications obtained from different lenses.

## 3. Results

### 3.1. Analysis of the Bone Graft Materials

#### 3.1.1. XRF

**Figure 1:**
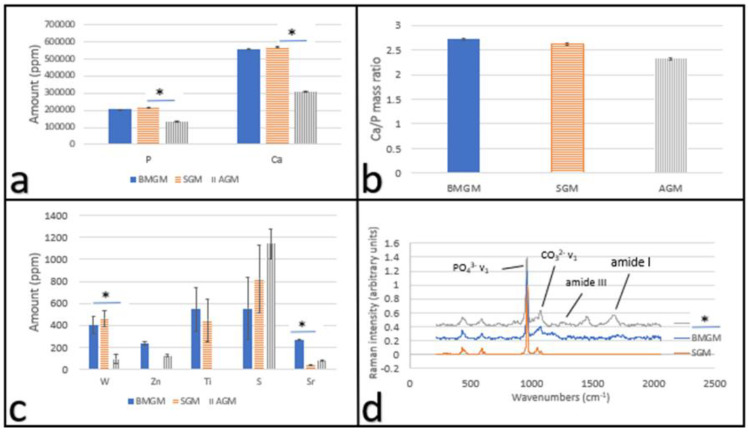
Characterization of bone graft materials: X-ray fluorescence of the bone chip samples (**a**) Hydroxyapatite: Ca and P amounts and (**b**) Ca to P ratio; (**c**) other trace metal impurities; * indicates *p* << 0.01 and (**d**) Raman spectra of the bone chip samples showing the amine and hydroxy apatite peaks.

In Figure 1a–b, we plot the Ca and P amounts and the Ca/P ratio from the XRF analysis of the materials tested. From Figure 1b, we find that the ratios are similar between the samples. From the XRF, we also found a small but statistically significant number of other impurities in the samples, which are plotted in Figure 1c. From the figure, we see that among these impurities, none of them have reported toxicities to human cells at the ppm levels, except for tungsten, where studies have shown that it is toxic at concentrations above 184 ppm [18]. From Figure 1c, we find that both the bovine (BMGM) and the synthetic (SGM) bone chips significantly exceed this amount, at 400 ± 77 ppm and 463 ± 73 ppm, respectively, while the human bone chips are significantly below this value, with 92 ± 42 ppm.

#### 3.1.2. Raman

In Figure 1d, we show Raman spectra from the three different materials used. From the spectra, we find the PO_4_^3−^ v_1_ symmetric stretch near 960 cm^−1^ and the substituted CO_3_^2−^ v_1_ in-plane vibrations near 1070 cm^−1^, corresponding to crystalline hydroxyapatite (HA) Ca_10_(PO_4_)_6_(OH)_2_, where the OH^−^ and/or PO_4_^3−^ groups can be substituted by carbonated CO_3_^2−^, and are present in all spectra. The spectrum of SGM is completely devoid of other peaks indicating that it is mostly HA. Even though some amide peaks are found in the spectrum of BMGM, their intensity is low, and they do not correspond to amide peaks expected for the collagenous matrix of mineralized tissue. Amide I mode (1595–1720 cm^−1^) and amide III envelope (1243–1269 cm^−1^) correspond to collagen, though they are only found in the spectrum of AGM. The mineral to protein ratio (PO_4_^3−^ v_1_ to amide I) of AGM is 3.55 ± 0.45, which is comparable to 3.81 ± 0.42 reported for human bone [19].

### 3.2. Tissue Culture Plastic (TCP) Substrate

#### 3.2.1. Dental Pulp Stem Cells

**Figure 2:**
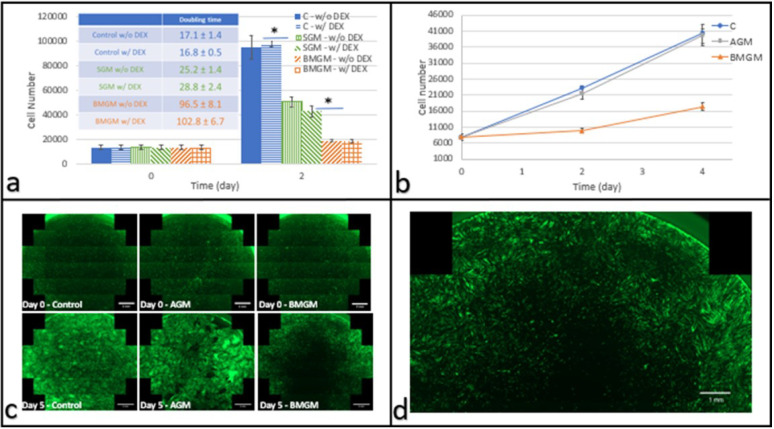
DPSCs cultured with bone graft materials on tissue culture plastic: (**a**) Cell numbers on days 0 and 2 after plating with and without DEX (*n* = 3) Inset: Corresponding doubling times (hours), with and without DEX. (**b**) Cell counts as a function of time in culture (w/o DEX) where * indicates *p* << 0.01 (**c**) fluorescence images of the entire well of eGFP DPSC (scale bar: 2 mm) at days 0 and 5 after addition of AGM or BMGM; Control wells received no additions. (**d**) Magnified fluorescence image of the well containing cells cultured with BMGM for 5 days (scale bar: 1 mm) showing a zone of exclusion around the region where the bone grafts were placed.

DPSCs were plated on TCP with and without BMGM and SGM bone chips. Cells were cultured with and without dexamethasone; the steroid was frequently added to promote osteogenic differentiation. The cell counts after two days, and the corresponding doubling times are shown in Figure 2a. From the figures, we can see that DEX did not impact the doubling times. On the other hand, the addition of the BMGM and SGM bone chips increased the doubling times from 16.8 ± 0.5 h to 96.5 ± 8.1 and 25.2 ± 1.4 h, respectively (*p* < 0.01). To probe whether the increased doubling times correlated with the tungsten impurities, we also measured the proliferation rate of culture with added AGM. The cell counts are plotted in Figure 2b together with those from culture using BMGM. From the figure, we can see that while the culture with BMGM was growing slowly, the proliferation rate in the culture with AGM was indistinguishable from the control.

In Figure 2c, we show the combined panoramic images of the entire DPSC culture wells corresponding to the control cells and those with added AGM and BMGM. From the figure, we can see that most cells were well extended in the control and the culture with added AGM, with both cultures showing increased cell density with time across the entire well. In contrast, in the culture with added BMGM, a large dark area is seen in the middle surrounding the area where a sliver of BMGM had been placed. A magnified view of cells cultured with BMGM after five days is seen in Figure 2d. From the figure, we can see that the cells initially adhered across the surface, but after five days, cell density near the BMGM was reduced with increases limited to regions furthest from the BMGM. Hence the cells away from the bone additives were proliferating normally along the periphery of the well and probably responsible for the upturn in cell counts with incubation time observed for the BMGM. These images are therefore consistent with the factors impacting cell proliferation as being leached slowly out from the added bone chips.

#### 3.2.2. Osteosarcoma Cells

**Figure 3:**
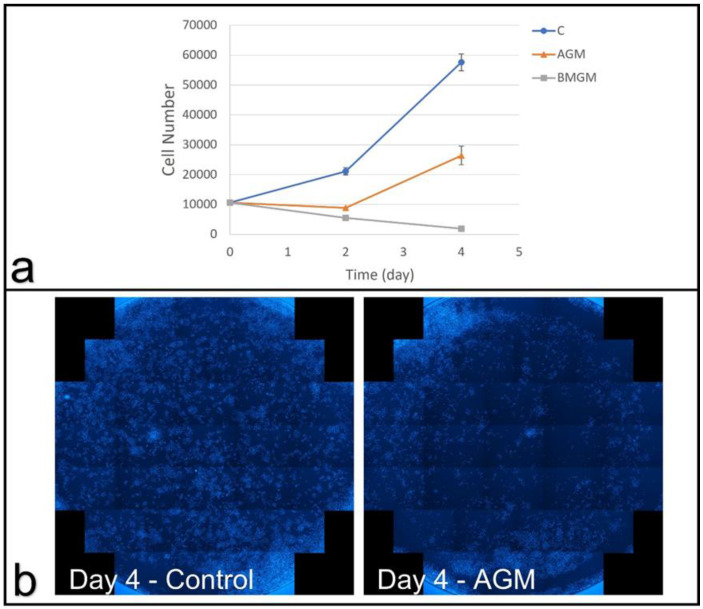
Saos-2 cells cultured with bone graft materials on TCP: (**a**) growth curves, and (**b**) fluorescence images of DAPI stained cell nuclei for the whole well showing the control group, without bone graft materials, and the group with AGM.

To determine the influence of the bone chips on osteoblasts, we also exposed Saos-2 cells to the same concentration of BMGM and AGM. The cell counts were plotted as a function of incubation time in Figure 3a, where we can see that during the first two days of exposure to BMGM or AGM, Saos-2 cultures showed cell loss. From day two to day four, cultures exposed to AGM proliferated while those exposed to BMGM continued to lose cells. The fluorescence images at day four are shown in Figure 3b, where a zone of exclusion, similar to the one observed for DPSC, is seen here as well. We, therefore, conclude that the Saos-2 cells are far more sensitive to the impurities in the bone chips than the DPSCs.

#### 3.2.3. Conditioned Medium

**Figure 4:**
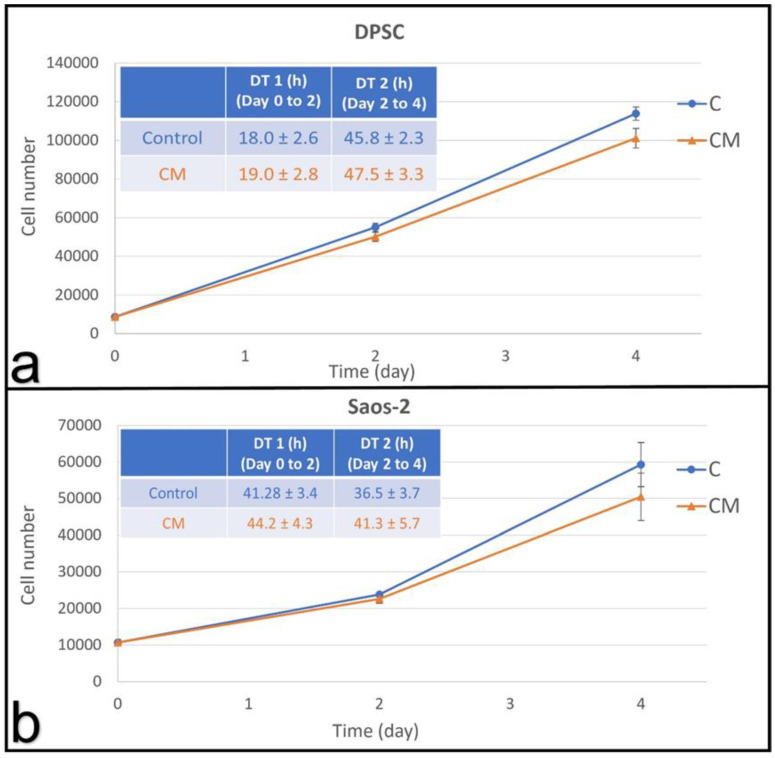
Growth curves and doubling times of cells cultured with conditioned media (CM) and control, without conditioned media (C)): (**a**) DPSCs and (**b**) Saos-2 cells.

In order to determine whether the observed toxicity was the result of factors released from the bone chips and stable in media, DPSC and Saos-2 growth in CM was measured. The cell counts of DPSCs as a function of incubation time are plotted in Figure 4a, where we find that no impact on the cell proliferation occurred when incubated in the conditioned media. This is consistent with the fluorescence images shown previously in Figure 2c,d, where a zone of inhibition was observed around the bone chips, which only extended a few millimeters but did not even reach the edges of the well. Furthermore, the Saos-2 cells, with a growth curve plotted in Figure 4b, which were even more sensitive to the bone chips, were not impacted by the conditioned medium, where the dilution further decreased the concentration below any the levels where toxicity was expected.

### 3.3. Collagen Substrates

#### 3.3.1. DPSC on Collagen

**Figure 5:**
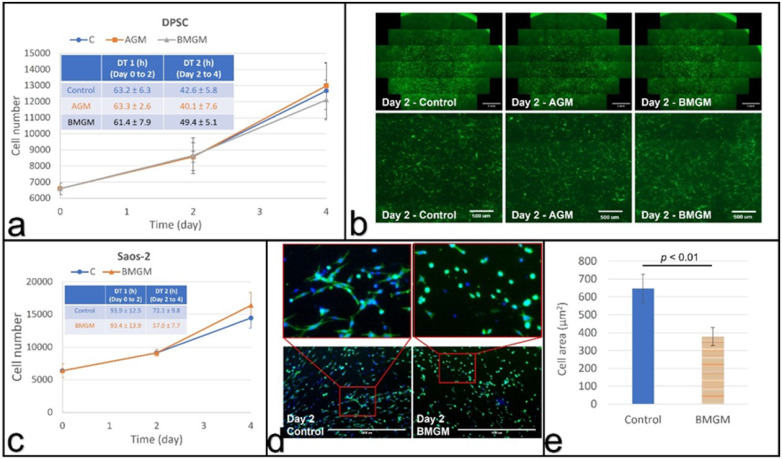
Cells cultured with bone graft materials on collagen substrates: (**a**) growth curves and calculated (inset) doubling times of DPSCs; (**b**) fluorescence images of DPSCs with the bottom row (scale bar: 500 µm) being magnified view of the area in the square shown in top row (scale bar: 2 mm); (**c**) growth curves and doubling times of Saos-2 cells; (**d**) fluorescence images of Saos-2 cells with the top row (scale bar: 1000 µm) being magnified view of the bottom row; (**e**) Comparison of the average cell area of Saos-2 cells at day 2.

In vivo, collagen is the most abundant component of the extracellular matrix for both DPSC and Saos-2. We, therefore, exposed both types of cells to BMGM to determine if toxicity persisted under those conditions as well. In Figure 5a, we show the growth curves and doubling times of the DPSCs cultured on collagen matrices in the presence of AGM and BMGM, as well as the control. From the figure, we can see that while the doubling times are longer for all samples on collagen, no significant difference can be discerned between the control culture or those with both types of bone chips. From the fluorescence images shown on the first row in Figure 5b, we also see that the cell density and extent of coverage are uniform across all samples. On the second row in Figure 5b, we plot magnified sections, where we can see that the cells are well extended and no differences in morphology is observed.

#### 3.3.2. Saos-2 on Collagen

Saos-2 cells were also cultured on collagen substrates, and the cell counts are plotted as a function of time in Figure 5c for the control and the sample containing BMGM. From the figure, we find that, despite the longer doubling time relative to TCP for the control, the counts are nearly identical for the first two days-in sharp contrast with the cultures plated on TCP, where no proliferation for the Saos-2 cells was observed.

Even though the level of significance is low, the cell counts in the culture containing BMGM appear to decrease between days two and four. We, therefore, examined the morphology of the cells on day two, and the images are shown in Figure 5d. From the figure, we can clearly see that the cells containing BMGM are significantly more contracted than the cells in the control samples on collagen. Cell extension is critical for achieving the proper cell tension in proliferation. The area of the cells is plotted in Figure 5e, where we can see that the value for the cells containing BMGM is significantly smaller. Hence the level of toxicity on the collagen substrates is greatly reduced if not eliminated.

## 4. Discussion

Bone chips are commonly used following multiple implant procedures, where the chips are obtained from multiple sources—synthetic, animal, or human. Sequestration of tungsten in bone has been demonstrated by numerous sources [13,20,21,22], but without concern, since the general, long-held assumption has been that it does not have any in vivo systemic toxicological impact. Recent findings have demonstrated that this assumption was not correct, and tungsten does accumulate systemically, especially in the vertebrae, where it is the source of increased inflammation and production of matrix metalloproteinases, eventually leading to disc failure [23]. Hence careful monitoring of tungsten in products implanted internally, where the tungsten can reach out and be transported systemically, should be a matter of concern and warrants close monitoring.

Analysis of a variety of bone chips indicated that both synthetic and animal samples contained tungsten at concentrations higher than 400 ppm. Much lower concentrations, 92 ppm, were found in human bone chips. Additionally, Raman showed that all products were composed of crystalline HA, but only the human bone chips also contained clearly identifiable amount of matrix proteins, where the mineral to matrix ratio, 3.55 ± 0.45, was similar to that found in bone, 3.81 ± 0.42 (*p* > 0.05).

Here we show that these tungsten concentrations found in the bone chips are indeed highly toxic when the bone chips have large concentrations when directly exposed to both human DPSC and Saos-2 cancerous osteoblasts. When cultured on tissue culture plastic, DPSC appeared to have a higher tolerance to the presence of the chips. After an initial decrease, their growth rate appeared to recover. Yet, a detailed examination of the EVOS images of the DPSC cultures showed large areas in the center, where the bone chips were placed were devoid of cells, even after two media changes. The cell numbers which were increasing were mainly on the periphery of the well. This indicated that W was probably leaching from the bone chips, killing cells in immediate proximity with the chips. With distance from the chips, the tungsten concentration decreased to levels below toxicity in the periphery of the wells. No effect on either proliferation or morphology was observed on the cultures grown in the presence of the human-derived bone chips-AGM. In this case, the cell counts were homogenous across the samples regardless of proximity to the bone chips.

The Saos-2 cells appeared to be even less tolerant to tungsten than the DPSC when cultured on tissue culture plastic. Saos-2 cells were unable to proliferate, and the cell counts decreased steadily between days two and four in culture on all samples, including those with only 94 ppm [17].

Bone chips are added to promote osseointegration into the surrounding bone via migration and adhesion of the host cells. These results indicate that the high levels of tungsten in the chips may be a cause for hindrance of the integration process and hence be responsible for possible failure.

In order to further explore the impact of the bone chips, the cells were also cultured on collagen, which is a natural component of the bone ECM, fusing the same high tungsten concentration chips–BMGM. From imaging data, it was immediately apparent that the degree of toxicity was greatly reduced. As expected, the proliferation rate of the cultures, including that of the control sample, was significantly reduced as compared to the cultures on TCP, but no discernable effects were observed for DPSC cultured with BMGM either in proliferation rates or cell morphology. Saos-2 cells initially proliferated at the same rate as the control, but with increasing time, cell morphology became rounded, and a slight decrease in proliferation rate was observed after two days.

These results highlight the importance of collagen as a substrate and the importance of mimicking as closely as possible the culture environment in extrapolating in vitro to in vivo conclusion. Even though the chemical nature of the interaction of collagen and tungsten is not known, there is experimental evidence that collagen can contain as much as 6% (mole percentage) tungsten [23]. Furthermore, since bone seems to be the locus of deposition of tungsten following systemic exposure, it is possible that the tungsten becomes sequestered within the collagen matrix associated with the bone. As this matrix degrades in the bone chips, the tungsten is released, but when new collagen is present, such as the one present in the extracellular matrix of bone cells, it is reabsorbed. Hence the toxicity to the cells immediately on the collagen is minimized. However, as the in vivo experiments indicate, the accumulation of tungsten eventually does elicit toxicity, and hence its introduction internally in humans should be severely limited.

## 5. Conclusions

Using DPSC and Saos-2 cells as an in vitro test bed, we show that bone graft materials can be cytotoxic, where the degree of toxicity was much higher for the Saos-2 cells. XRF analysis indicated showed tungsten concentrations of 400 ± 77, 463 ± 73 and 92 ± 42 ppm in BMGM, SGM, and AGM, while Raman spectroscopy indicated the presence of collagen only in AGM with a mineral/protein ratio of 3.55 ± 0.45, consistent with human bone. Significant toxicity was observed on BMGM and SGM for all cells cultured on TCP. No toxicity was observed for AGM on DPSC, while initial toxicity was observed for Saos-2 by day two, followed by partial recovery by day four. Analysis of the fluorescence images shows zones of exclusion surrounding the graft materials with lower or no cell counts. These results are consistent with tungsten toxicity, where the sensitivity is higher for the Saos-2 than the DPSC. Plating both types of cells on collagen substrates mitigates the toxicity for all the materials, consistent with previous reports of tungsten adsorption into collagen. Furthermore, the cells in these experiments were subjected to low levels of particles. In vivo, the bone chips are tightly packed, and much higher concentrations may be present, emphasizing the caution with which these materials should be used in practice. These results were not expected but clearly highlight that research into dental materials should be an ongoing process.

## Data Availability

Not Applicable.
